# Expression of transcription factor AP-2α predicts survival in epithelial ovarian cancer

**DOI:** 10.1054/bjoc.2000.1146

**Published:** 2000-05-18

**Authors:** M A Anttila, J K Kellokoski, K I Moisio, P J Mitchell, S Saarikoski, K Syrjänen, V-M Kosma

**Affiliations:** Departments of Obstetrics and Gynecology, Pathology and Forensic Medicine, Pathology, Oncology, Oral and Maxillofacial Unit, University of Kuopio and Kuopio University Hospital, PO Box 1627, Kuopio, FIN-70211, Finland; Department of Biochemistry & Molecular Biology, Pennsylvania State University, University Park, South Frear, PA, USA

**Keywords:** epithelial ovarian cancer, prognosis, AP-2α, p21/WAF1

## Abstract

The 52-kDa activator protein (AP)-2 is a DNA-binding transcription factor which has been reported to have growth inhibitory effects in cancer cell lines and in human tumours. In this study the expression of AP-2α was analysed in 303 epithelial ovarian carcinomas by immunohistochemistry (IHC) with a polyclonal AP-2α antibody and its mRNA status was determined by in situ hybridization (ISH) and reverse transcriptase-polymerase chain reaction (RT-PCR). The immunohistochemical expression of AP-2α was correlated with clinicopathological variables, p21/WAF1 protein expression and survival. In normal ovaries, epithelial cells expressed AP-2α protein only in the cytoplasm. In carcinomas nuclear AP-2α expression was observed in 28% of the cases although cytoplasmic expression was more common (51%). The expression of AP-2α varied according to the histological subtype and differentiation. AP-2α and p21/WAF1 expressions did not correlate with each other. Both in univariate (*P* = 0.002) and multivariate analyses (relative risks (RR) 1.6, 95% confidence interval (CI) 1.13–2.18, *P* = 0.007) the high cytoplasmic AP-2α expression favoured the overall survival. In contrast, the nuclear AP-2α expression combined with low cytoplasmic expression increased the risk of dying of ovarian cancer (RR = 2.10, 95% CI 1.13–3.83, *P* = 0.018). The shift in the expression pattern of AP-2α (nuclear vs cytoplasmic) in carcinomas points out to the possibility that this transcription factor may be used by oncogenes in certain histological subtypes. Based on the mRNA analyses, the incomplete expression and translation of AP-2α in ovarian cancer may be due to post-transcriptional regulation. © 2000 Cancer Research Campaign


*British Journal of Cancer* (2000) **82**(12), 1974–1983
© 2000 Cancer Research Campaign
doi: 10.1054/ bjoc.2000.1146, available online at http://www.idealibrary.com on IDEAL®

### Expression of transcription factor AP-2α predicts survival in epithelial ovarian cancer

**MA Anttila[1,2], JK Kellokoski[2,4,5], KI Moisio[2], PJ Mitchell[6], S Saarikoski[1], K Syrjänen[2,3] and V-M Kosma[2,3]**

Departments of [1]Obstetrics and Gynecology, [2]Pathology and Forensic Medicine, [3]Pathology, [4]Oncology and [5]Oral and Maxillofacial Unit, University of Kuopio and Kuopio University Hospital, PO Box 1627, FIN-70211 Kuopio, Finland; [6]Department of Biochemistry & Molecular Biology, Pennsylvania State University, University Park, South Frear, PA, USA

**Summary** The 52-kDa activator protein (AP)-2 is a DNA-binding transcription factor which has been reported to have growth inhibitory effects in cancer cell lines and in human tumours. In this study the expression of AP-2α was analysed in 303 epithelial ovarian carcinomas by immunohistochemistry (IHC) with a polyclonal AP-2α antibody and its mRNA status was determined by in situ hybridization (ISH) and reverse transcriptase-polymerase chain reaction (RT-PCR). The immunohistochemical expression of AP-2α was correlated with clinicopathological variables, p21/WAF1 protein expression and survival. In normal ovaries, epithelial cells expressed AP-2α protein only in the cytoplasm. In carcinomas nuclear AP-2α expression was observed in 28% of the cases although cytoplasmic expression was more common (51%). The expression of AP-2α varied according to the histological subtype and differentiation. AP-2α and p21/WAF1 expressions did not correlate with each other. Both in univariate ($P$ = 0.002) and multivariate analyses (relative risks (RR) 1.6, 95% confidence interval (CI) 1.13–2.18, $P$ = 0.007) the high cytoplasmic AP-2α expression favoured the overall survival. In contrast, the nuclear AP-2α expression combined with low cytoplasmic expression increased the risk of dying of ovarian cancer (RR = 2.10, 95% CI 1.13–3.83, $P$ = 0.018). The shift in the expression pattern of AP-2α (nuclear vs cytoplasmic) in carcinomas points out to the possibility that this transcription factor may be used by oncogenes in certain histological subtypes. Based on the mRNA analyses, the incomplete expression and translation of AP-2α in ovarian cancer may be due to post-transcriptional regulation. © 2000 Cancer Research Campaign

**Keywords:** epithelial ovarian cancer; prognosis; AP-2α; p21/WAF1

The 52-kDa activator protein (AP)-2 is a DNA-binding transcription factor, which was originally purified from HeLa cells and identified as a nuclear factor, regulating the expression of human metallothionein IIa (Lee et al, 1987; Williams et al, 1988). AP-2 also mediates programmed gene expression during embryogenesis including genital tract, and cell differentiation in adult tissues (Williams et al, 1988; Mitchell et al, 1991). The AP-2 amino acid sequence is highly conserved in mice and humans indicating that this protein plays a fundamental role in development (Winning et al, 1991). Transcriptionally AP-2 functions as a dimer and recognizes GC-rich DNA-sequences (Williams et al, 1988). The DNA-binding domain is located within the C-terminal half of the protein, showing high conservation (75–80%) within three human AP-2 related genes AP-2α, -β and γ (Bosher et al, 1996; Williamson et al, 1996).

In normal cells, AP-2 regulates gene expression in response to different signal transduction pathways. Signals mediated through the activation of cAMP-dependent protein kinase and protein kinase C induce AP-2 activity independent of protein synthesis (Chiu et al, 1987; Imagawa et al, 1987; Hyman et al, 1989). In contrast, retinoic acid induces AP-2 activity by increasing AP-2 mRNA levels (Williams et al, 1988; Luscher et al, 1989). AP-2 activates both in vivo and in vitro transcription of promoters containing AP-2 binding sites, which have been identified in the enhancer regions of viral genes (Mitchell et al, 1987), proenkephalin, keratin K14 (Leask et al, 1991), c-*erb*B-2 (Bosher et al, 1995), plasminogen activator type I, c-*Myc* (Imagawa et al, 1987), E-cadherin (Hennig et al, 1996), type IV collagenase (MMP-2) (Huhtala et al, 1990) and p21/WAF1 (Zeng et al, 1997). Recently, it was reported that transcriptional stimulation of E-cadherin promoter by pRb and Myc is mediated by AP-2 in epithelial cells (Batsche et al, 1998).

The AP-2α, -β and -γ genes are located at chromosomes 6p24, 6p12 and 20q13 respectively (Williamson et al, 1996). Loss of heterozygosity has been frequently reported on chromosome 6p in human cancers, including ovarian cancer (Sato et al, 1991; Foulkes et al, 1993). In cancer cell lines (Bar-Eli, 1997; Zeng et al, 1997; Jean et al, 1998) and in malignant melanoma (Karjalainen et al, 1998) AP-2 has been shown to possess or exert growth inhibitory effects. The tumour suppressive effects of AP-2 are, at least in part, mediated through p21/WAF1 activation (Zeng et al, 1997). To explore the significance of AP-2 in ovarian cancer, we studied the expression of AP-2α in 303 epithelial ovarian carcinomas using IHC. Furthermore, the relationships between AP-2α and p21/WAF1 expressions, histopathological variables and patient survival were analysed. To further elucidate the regulatory mechanisms, the mRNA status of AP-2α was studied by RNA ISH and confirmed by RT-PCR.

### MATERIALS AND METHODS

### Patients

The material of the present study was selected from a consecutive series of 445 women diagnosed and treated for ovarian malignancy

*Received 2 June 1999*
*Revised 15 November 1999*
*Accepted 17 January 2000*

*Correspondence to:* MA Anttila, Department of Pathology and Forensic Medicine, University of Kuopio, PO Box 1627, FIN-70211 Kuopio, Finland at Kuopio University Hospital and Jyväskylä Central Hospital, Finland, between 1976 and 1992 and subsequently followed-up until September 1996. The non-epithelial type of neoplasia and all patients treated before operation or unoperated patients were excluded. Depending on the availability of representative tumour samples, a total of 303 patients were included in the analyses.

All tumours were staged according to the International Federation of Gynecology and Obstetrics (FIGO) standards (Cancer Committee of the International Federation of Gynecology and Obstetrics, 1989). Retrospective review of the patient files was performed to obtain all pertinent data on the primary tumour, type of surgery, adjuvant treatment, recurrence and survival. Patients who died because of any post-operative complications were excluded from the survival analyses ($n$ = 9). The clinicopathological data of the patients are summarized in Table 1.

Of the 303 patients, 120 underwent radical surgery (i.e. no primary residual tumour), 221 received post-operative chemotherapy, nine received post-operative radiotherapy and 38 women received both therapies. Disease recurrence was observed in 73 patients (24%), no recurrence in 92 patients (30%) and in 138 patients (46%) the tumour was present or progressing. The median follow-up time for all patients ($n$ = 303) was 27 months (range 1–237 months) and for patients still alive ($n$ = 77) 106 months (range 22–237 months).

**Table 1** Clinical data of the patients

| **Age at diagnosis, years** | **Median**<br>**Range** | **62**<br>**18–85** | |
|---|---|---|---|
| Histological type | epithelial | *n* | % |
| | serous | 107 | 35 |
| | mucinous | 30 | 10 |
| | endometrioid | 82 | 27 |
| | clear cell | 31 | 10 |
| | other[a] | 53 | 18 |
| Histological grade | 1 | 41 | 13 |
| | 2 | 105 | 35 |
| | 3 | 157 | 52 |
| FIGO stage | I | 83 | 27 |
| | II | 46 | 15 |
| | III | 144 | 48 |
| | IV | 30 | 10 |
| Primary residual tumour | no data | 26 | 8 |
| | none | 120 | 40 |
| | ≤2 cm | 51 | 17 |
| | >2 cm | 106 | 35 |
| Adjuvant chemotherapy | platinum-containing | 160 | 53 |
| | no platinum-containing | 96 | 32 |
| | none | 44 | 15 |
| Chemotherapy response[b] | no data | 7 | 2 |
| | CR | 138 | 45 |
| | PR | 39 | 13 |
| | SD | 21 | 7 |
| | PD | 54 | 18 |
| Cause of death | ovarian cancer | 195 | 65 |
| | other cause | 31 | 10 |
| | alive | 77 | 25 |

[a]includes 20 mixed epithelial, one Brenner, 32 unclassified epithelial.
[b]CR = complete response, PR = partial response, SD = stable disease, PD = progressing disease, no chemotherapy 44 (15%).

### Histology

The primary tumour samples were fixed in 10% formalin and embedded in paraffin. From each sample, 5-µm-thick sections were stained with haematoxylin and eosin. Histological typing and grading were done according to the WHO classification (Serov et al, 1973). All tumours were graded as either well, moderately or poorly differentiated by one pathologist (KS) in a blinded manner, i.e. being unaware of the other data.

### Immunohistochemistry

### *AP-2α*

Five-micrometre-thick tumour sections were deparaffinized and rehydrated using xylene and graded alcohols. Endogenous peroxidase activity was blocked by 5% hydrogen peroxide for 5 min, followed by a wash for 2 × 5 min with phosphate-buffered saline (PBS). The sections were heated in a microwave oven in 10 mM citrate buffer (pH 6.0) for 2 × 5 min and after that allowed to cool for 20 min and washed for 3 × 5 min with PBS. Non-specific binding was blocked with 1.5% normal goat serum in PBS for 30 min at 37°C. The primary antibody, a rabbit polyclonal AP-2α (SC-184) (Santa Cruz Biotechnology, Santa Cruz, CA, USA) antibody, raised against a peptide corresponding to amino acids 420–437 mapping at the carboxy terminus of AP-2 of human origin, was diluted to 1:2500 and incubated on the slides overnight at 4°C. The slides were incubated with the secondary antibody for 30 min and washed for 2 × 5 min with PBS. The slides were then incubated for 40 min in preformed avidin–biotinylated peroxidase complex (ABC, Vectastain Elite kit, Vector Laboratories, Burlingame, CA, USA) and washed twice for 5 min with PBS, developed with diaminobenzidine tetrahydrochloride (DAB) substrate (Sigma, St Louis, MO, USA), counterstained with Mayer's haematoxylin, dehydrated, cleared and mounted with DePex (BDH, Poole, UK). A corresponding section processed without the primary antibody was used as a negative control and the inflammatory cells within the tumour served as positive internal controls.

### *p21/WAF1*

The p21/WAF1 protein was demonstrated using a similar IHC staining protocol. We used p21-specific mouse monoclonal antibody (NCL-WAF-1, Novocastra Laboratories Ltd, Newcastle upon Tyne, UK) at a working dilution of 1:10. In each batch, known p21-positive tumour samples were used as positive controls, and the same biopsy processed without the primary antibody was used as a negative control.

### *Scoring of the AP-2 and p21/WAF1 immunostainings*

All slides were evaluated with a microscope (field diameter 490 µm) and the fraction of positive cancer cells was assessed as the percentage of the whole tumour area by two observers (MA, V-MK) being unaware of the clinical outcome of the patients. Discrepancies between the observers were found in fewer than 10% of the slides examined, and consensus was reached on further review. For AP-2α, both the nuclear and cytoplasmic positivity was recorded. The nuclear AP-2α expression was graded into two groups (negative or positive) according to 5% cut-off point, which was the 75th percentile in a frequency distribution. The cytoplasmic AP-2α expression was graded into two groups according to 50th percentile in a frequency distribution:

low, when < 10% of tumour cells expressed AP-2α and high, when ≥ 10% of tumour cells expressed AP-2α. For p21, the positivity was assessed as the percentage of positively stained tumour cell nuclei. For further analyses, p21/WAF1 immunoreactivity was categorized into two groups: (1) low expression, if < 10% of the tumour cell nuclei were positive, and (2) high expression, if ≥ 10% of the tumour cell nuclei were positive (Ito et al, 1996; Wakasugi et al, 1997). The 10% cut-off point was the 75th percentile of the p21/WAF1 expression in a frequency distribution.

### mRNA in situ hybridization

A series of 19 samples (both AP-2α-negative and -positive by IHC) being representative of the tumour material were selected for ISH. Deparaffinized 5-µm sections were microwaved in 0.01 M citrate buffer (pH 6.0) for 5 min. The samples were fixed in 4% paraformaldehyde, rinsed, pretreated with 0.1 M hydrochloric acid (HCl) and PBS, followed by digestion for 20 min with proteinase K (Boehringer Mannheim, Mannheim, Germany) at the concentration of 20 µg $ml^{-1}$. After a short fixation by 4% paraformaldehyde, the sections were rinsed in PBS and in 0.85% sodium chloride (NaCl), dehydrated in graded ammonium acetate alcohols and air-dryed. All specimens were then prehybridized for 2 h at 62°C. Recombinant plasmid pHAP2-Hsma containing human AP-2α exon 2 (transactivation domain) in a pBluescript KS+ -vector was linearized to generate antisense- and sense-digoxigenin-labelled probes by DIG RNA labelling kit (Boehringer Mannheim). Probes were added to the hybridization buffer at 5 ng $\mu l^{-1}$, and the sections were hybridized overnight at 62°C in a humified chamber. Subsequently, the sections were washed with increasing stringency. Non-specific binding was blocked with 2% normal sheep serum (Sigma) in 0.1% Triton X/TBS-buffer. The hybridized probe was detected by incubation in anti-digoxigenin alkaline phosphatase conjugate (Boehringer Mannheim) followed by NBT/BCIP. The reaction was stopped with diluted water, sections were dehydrated and coverslipped with Mountex (Histolab Production AB, Göteborg, Sweden). The specificity of the hybridization signals was demonstrated with digestion of the serial section with RNAase before microwave or proteinase K pretreatment to degrade RNA and secondly, a consecutive section was stained with the sense cRNA probe labelled to the same specific activity as the corresponding antisense probe. The intensity of ISH staining was graded as negative (0), weak (1), moderate (2) or strong (3) (Sperry et al, 1996).

### RT-PCR amplification

To confirm the specificity of the AP-2α mRNA ISH signal, total cellular RNA was extracted from five 20-µm-thick paraffin-embedded tissue sections of the tumour as described previously (Sugg et al, 1998). The RNA was used as a template to synthesize first-strand cDNA, and PCR amplification was performed using the commercially available Enhanced Avian RT-PCR kit (Sigma). The primers for the AP-2α were designed to anneal to exon 2 of the TFAP2A gene and their specificity was checked by searching the Blast database. The primers used were: AP-2 U 5′-GCCCCGT-GTCCCTGTCCAA-3′ and AP-2 L 5′-TGAGGAGCGAGAGGC-GACC-3′. PCR products were electrophoresed on a 6% poly-acrylamide gel and stained with silver nitrate.

### Statistical analyses

The SPSS-Win 7.5 program package was used in a PC computer for basic statistical calculations. First, the relationships (Spearman correlations) between AP-2α and p21 expression levels were analysed. Each parameter in the analysis was considered as a continuous variable. The interrelationships between the categorical IHC variables and their associations with clinicopathological parameters were examined by contingency tables which were further analysed by $\chi^2$-tests. Univariate survival analyses were based on the Kaplan–Meier method (log- rank analysis) (Kaplan and Meier, 1958). Univariate and multivariate survival analyses were calculated with Cox model using the Log likelihood ratio significance test in a forward stepwise manner (Cox, 1999). Overall survival was defined as the time interval between the date of surgery and the date of death due to ovarian cancer. Recurrence-free survival was defined by the time interval between the date of surgery and the date of diagnosed recurrence. Probability values less than 0.05 were regarded as significant. In Cox's multivariate analysis, a removal limit of $P < 0.10$ was used as an additional criteria.

### RESULTS

### AP-2α expression

In immunohistochemical analyses of AP-2α protein, negative control section remained negative and in tumour sections, strongly positive inflammatory cells served as internal positive controls (Figure 1A). In normal ovarian surface epithelium ($n = 19$) no nuclear AP-2α expression was observed but high cytoplasmic AP-2α expression was observed in 63% of the cases.

Positive nuclear AP-2α expression was observed in 28% of the tumours (Figure 1B). Of the total ($n = 303$), 34% of serous, 34% of endometrioid and 42% of clear cell histological subtypes were AP-2α positive ($\chi^2 = 22.7$, $P = 0.0001$). The majority of the AP-2α positivity was expressed in the cytoplasm (Figure 1 C,D). Of the tumours, 51% were high expressors of cytoplasmic AP-2α including 70% of the mucinous and 65% of the clear cell tumours ($\chi^2 = 11.6$, $P = 0.02$). Both nuclear and cytoplasmic AP-2α positivities were observed in 19% of the tumours (Figure 1E).

The high cytoplasmic AP-2α expression associated with better differentiation ($\chi^2 = 6.1$, $P = 0.01$), absence of residual tumour ($\chi^2 = 5.7$, $P = 0.02$) and complete chemotherapy response group ($\chi^2 = 7.8$, $P = 0.05$). The nuclear AP-2α expression had no statistical correlation with the p21/WAF1 expression (Figure 1F).

### AP-2α mRNA expression

All tumours ($n = 19$) showed AP-2α mRNA positivity in the cytoplasm, the intensity being mostly moderate or strong (Figure 2A). Of these, nine were negative for nuclear AP-2α, one for cytoplasmic AP-2α and two were totally AP-2α protein-negative by IHC (Table 2). Negative control sections of each specimen remained negative throughout the ISH analyses (Figure 2B). Positive mRNA signal was also observed by ISH and RT-PCR (Figure 3) in those tumours that expressed only cytoplasmic and/or nuclear AP-2α by IHC.

### p21/WAF1 expression

A total of 298 ovarian tumours were evaluable for p21/WAF1 immunostaining which was confined to the tumour cell nuclei. Of

*© 2000 Cancer Research Campaign*

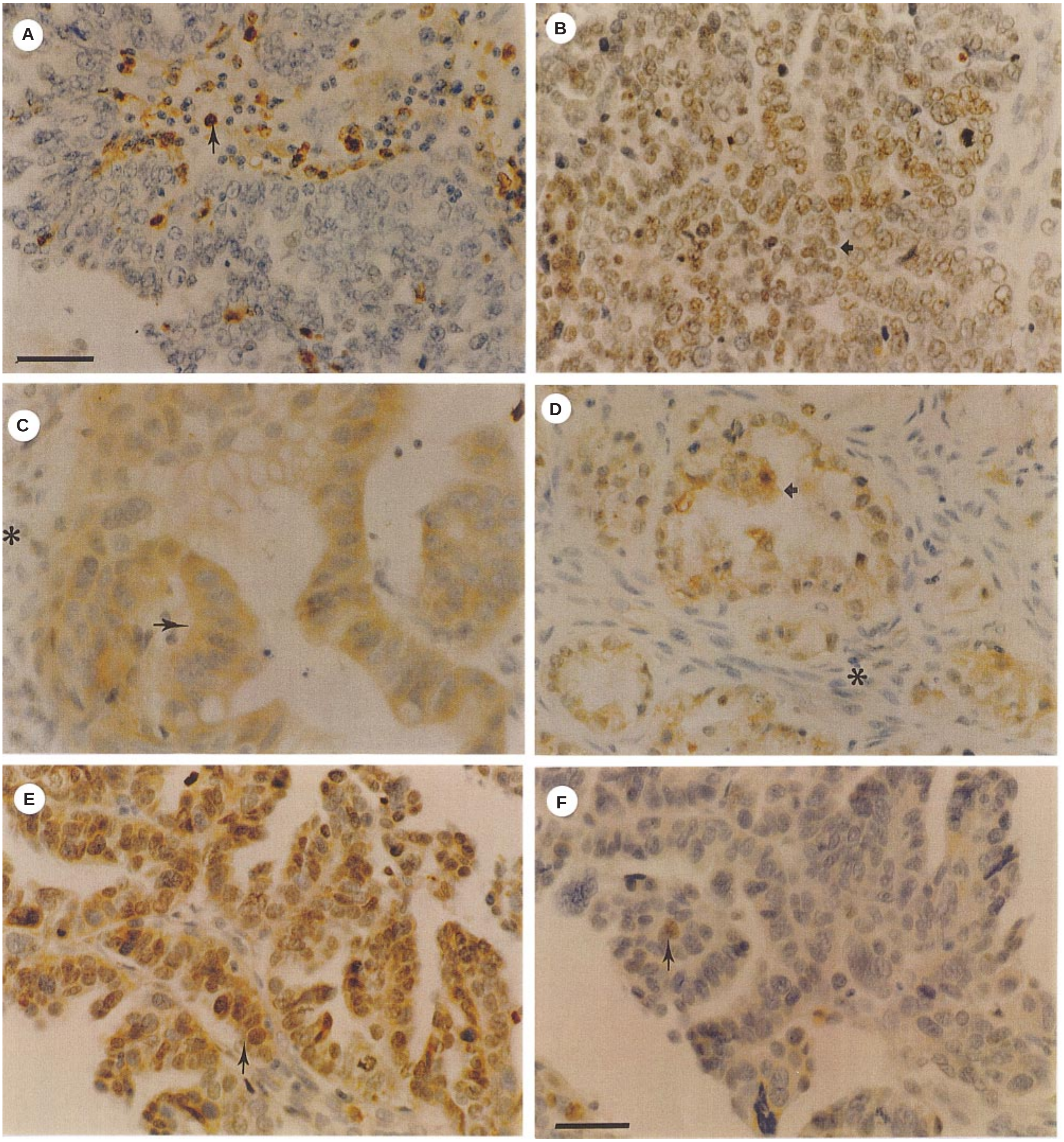

**Figure 1** AP-2α immunohistochemical analysis of (**A**) an endometrioid ovarian cancer with AP-2α negative tumour cells and AP-2α positive inflammatory cells (arrow), (**B**) a serous ovarian cancer with AP-2α positive nuclei (arrow), (**C**) a mucinous ovarian cancer showing a strong cytoplasmic expression of AP-2α (arrow) and negative interstitial tissue (asterisk), (**D**) a clear cell tumour with cytoplasmic AP-2α positivity (arrow); interstitial cells (asterisk) are AP-2α-negative, (**E**) a serous ovarian cancer showing positivity both in tumour cell nuclei and cytoplasm, and (**F**) the same tumour showing only few (arrow) p21/WAF1-positive tumour cell nuclei. Bar = 50 μm these, 206 tumours (69%) showed low expression of p21/WAF1. The low p21/WAF1 expression associated significantly with the serous and other histological types ($\chi^2 = 41.2$, $P < 0.000005$), poor differentiation ($\chi^2 = 14.2$, $P = 0.0001$), advanced FIGO stage ($\chi^2 = 9.0$, $P = 0.003$) and residual tumour > 2 cm ($\chi^2 = 13.1$, $P = 0.0003$).

### Univariate survival analysis

Overall disease-related 5-year survival rate of the patients was 37%. When nuclear and cytoplasmic AP-2α expressions were considered separately, nuclear AP-2α expression was not related

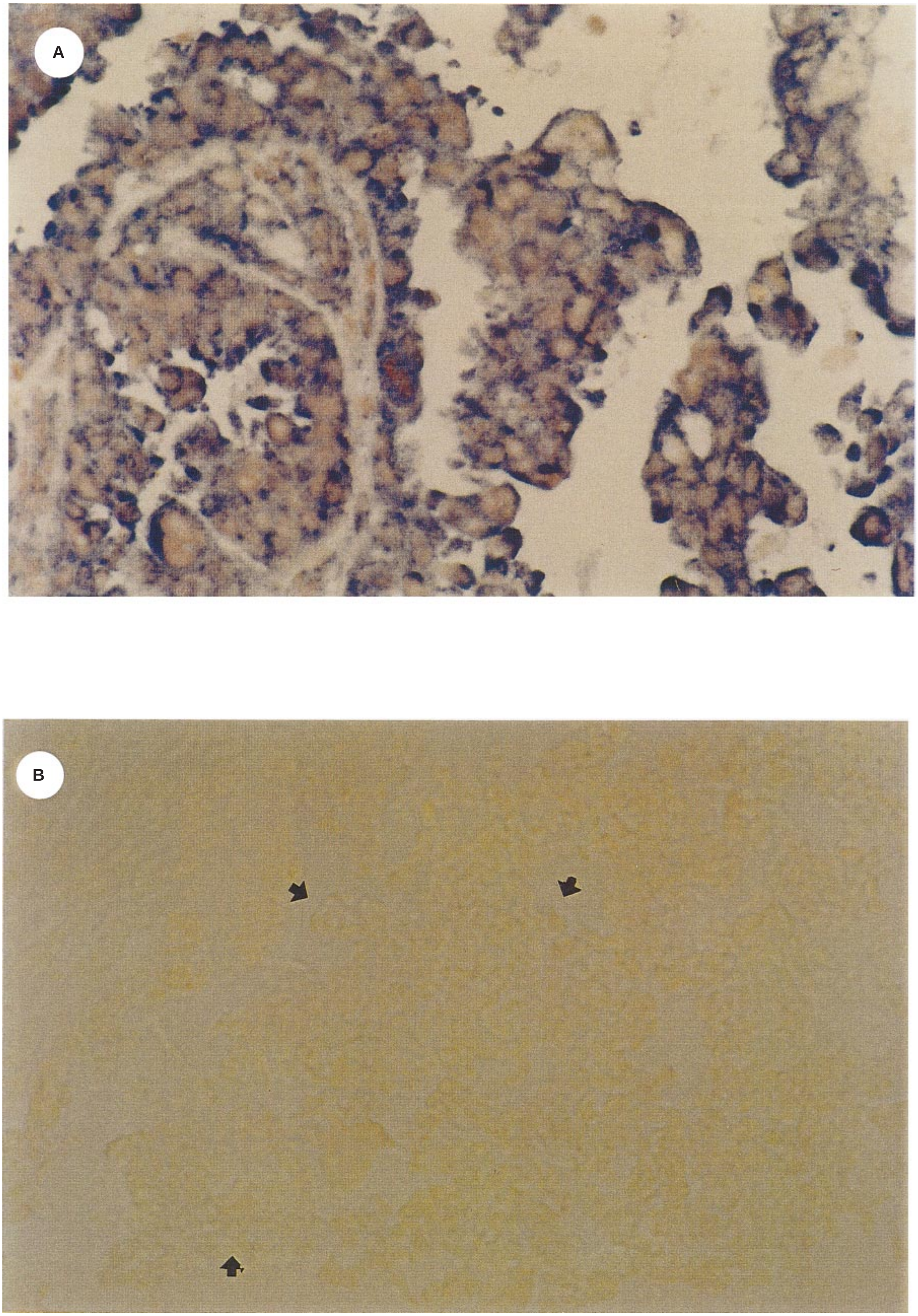

**Figure 2** (**A**) Positive mRNA in situ hybridization analysis for AP-2α and (**B**) a consecutive negative control section hybridized with the sense probe showing no positivity. Some of the tissue boundaries are marked by arrows

© 2000 Cancer Research Campaign

**Table 2** Results of the AP-2α mRNA in situ hybridization and immunohistochemical analyses

| No. | Histological type | Histological grade | FIGO stage | mRNA intensity[a] | Nuclear AP-2α positivity (%) | Cytoplasmic AP-2α positivity (%) | Cause of death | Follow-up time (months) |
|---|---|---|---|---|---|---|---|---|
| 1 | Serous | 2 | III | 3 | 40 | 90 | Alive | 41 |
| 2 | Serous | 3 | III | 2 | 60 | 0 | Ovarian Cancer | 26 |
| 3 | Serous | 3 | III | 3 | 0 | 0 | Other | 16 |
| 4 | Serous | 3 | I | 2 | 0 | 5 | Alive | 49 |
| 5 | Mucinous | 1 | I | 1 | 0 | <5 | Alive | 46 |
| 6 | Mucinous | 3 | I | 3 | 0 | 70 | Alive | 52 |
| 7 | Mucinous | 2 | I | 2 | 0 | 70 | Alive | 118 |
| 8 | Endometrioid | 3 | III | 3 | 0 | 5 | Ovarian Cancer | 6 |
| 9 | Endometrioid | 3 | I | 2 | 0 | 5 | Ovarian Cancer | 41 |
| 10 | Endometrioid | 2 | I | 2 | 40 | 40 | Other | 139 |
| 11 | Endometrioid | 2 | III | 2 | 50 | 40 | Ovarian Cancer | 10 |
| 12 | Clear cell | 1 | II | 3 | 40 | 60 | Ovarian Cancer | 5 |
| 13 | Clear cell | 2 | III | 3 | 10 | 20 | Ovarian Cancer | 1 |
| 14 | Clear cell | 2 | III | 3 | 5 | 5 | Ovarian Cancer | 14 |
| 15 | Clear cell | 2 | I | 3 | 40 | 50 | Ovarian Cancer | 29 |
| 16 | Clear cell | 3 | I | 2 | 60 | 50 | Ovarian Cancer | 98 |
| 17 | Other | 3 | III | 3 | <5 | 20 | Other | 30 |
| 18 | Other | 3 | I | 2 | 0 | 10 | Alive | 189 |
| 19 | Other | 3 | IV | 3 | 0 | 0 | Ovarian Cancer | 13 |

[a]1 = weak, 2 = moderate, 3 = strong.

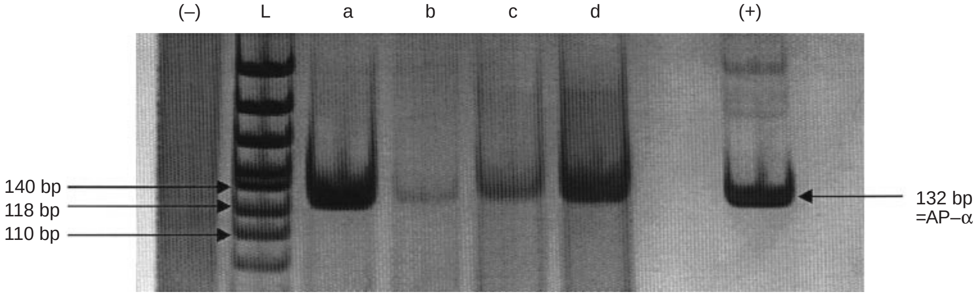

**Figure 3** Expression of AP-2α mRNA in epithelial ovarian cancer. Exon 2 region of the TFAP2A gene was analysed by RT-PCR on a 6% polyacrylamide gel. The far left (–) depicts a negative control in which a template was replaced by water ($H_2O$), (L) line contains a size ladder indicating molecular weight, (**A**), (**B**) and (**C**) are tumours with AP-2α-negative nuclei and positive cytoplasm, (**D**) is a tumour with both AP-2α-positive nuclei and cytoplasm, (+) is a positive control for AP-2α RNA (from Clontech Human Lung cDNA Library). All cases (**A–D**) contain PCR product of the same molecular size (132 bp) as the positive control sample to survival ($P = 0.45$) but the high cytoplasmic AP-2α expression predicted significantly favourable overall survival in univariate analysis (28% vs 46%, $P = 0.002$) (Figure 4). When both the nuclear and cytoplasmic AP-2α expressions were taken into account for univariate survival, the significant survival advantage was related to high cytoplasmic AP-2α expression without nuclear AP-2α ($P = 0.006$). Results of Cox's univariate survival analysis according to the AP-2α expression are presented in Table 3. Other significant predictors of favourable overall survival were early FIGO stage ($P < 0.00005$), differentiation ($P < 0.00005$), absence of primary residual tumour ($P < 0.00005$), younger age at diagnosis ($P = 0.0004$) and high p21/WAF1 expression ($P = 0.02$).

The high cytoplasmic AP-2α expression associated significantly with better overall survival also in the subgoups of endometrioid ($P = 0.008$), clear cell ($P = 0.045$) and other ($P = 0.03$) histological subtypes. The same association was observed in moderately ($P = 0.03$) and poorly ($P = 0.007$) differentiated tumours. For those patients with advanced FIGO stages high cytoplasmic AP-2α expression predicted better overall survival ($P = 0.01$).

*© 2000 Cancer Research Campaign* 

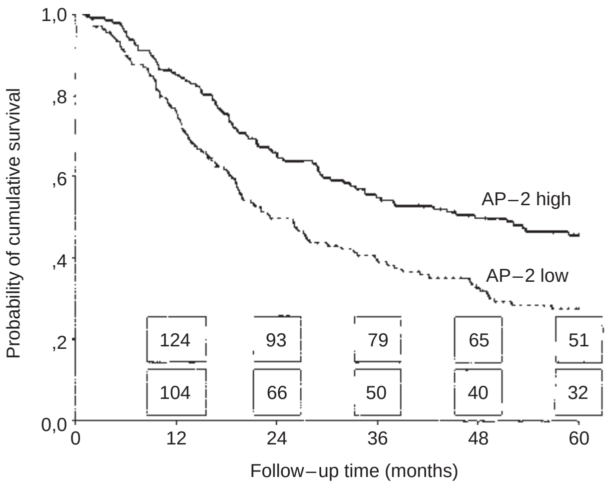

**Figure 4** Overall Kaplan–Meier survival analysis of the patients according to cytoplasmic AP-2α expression, $P = 0.002$ (log-rank analysis). Above the follow-up intervals the number of individuals available for censoring is presented (upper row, high AP-2α; lower row, low AP-2α)

Short recurrence-free survival was significantly predicted by serous histological type ($P = 0.01$), advanced FIGO stage ($P < 0.00005$) and primary residual tumour > 2 cm ($P < 0.00005$). AP-2α expression did not relate to the recurrence-free survival in Kaplan–Meier or in Cox's univariate survival analyses.

### Multivariate survival analysis

The histological type and grade, FIGO stage, age at diagnosis, primary residual tumour, adjuvant chemotherapy, p21/WAF1 and cytoplasmic AP-2α expression were included in the Cox's multivariate analysis (Table 4). The independent prognostic factors of overall survival were FIGO stage ($P = 0.0009$), primary residual tumour ($P = 0.0001$), age at diagnosis ($P = 0.009$), adjuvant chemotherapy ($P = 0.007$) and cytoplasmic AP-2 expression ($P = 0.007$). When combined nuclear and cytoplasmic AP-2α expression was included in the Cox analysis, the risk to die of ovarian cancer was even more increased if nuclear positivity was observed together with low cytoplasmic expression of AP-2α (RR = 2.10, 95% CI 1.13–3.83, $P = 0.018$). Only the primary residual tumour predicted independently recurrence-free survival ($P < 0.00005$).

### DISCUSSION

In this study we show that malignant epithelial ovarian tumours express transcription factor AP-2α in cytoplasm as well as in tumour cell nuclei compared to normal ovaries where only cytoplasmic staining was observed. The significance of AP-2α expression in survival analyses points out the importance of this transcription factor in epithelial ovarian malignancy, although the precise mechanisms of AP-2α's functions remain unknown.

In vitro, AP-2 is expressed in a cell-type-specific manner (Imagawa et al, 1987; Williams et al, 1988). So far, only few studies have examined the expression of AP-2 in human tumours (Gilbertson et al, 1997; Hietala et al, 1997; Karjalainen et al, 1998; Turner et al, 1998). In the present series, AP-2α expression was observed both in the cancer cell nuclei and cytoplasm, and the expression varied according to the histological subtype and differentiation. Both nuclear and cytoplasmic expression of AP-2α has been observed also in malignant melanomas (Karjalainen et al, 1998), childhood medulloblastomas (Gilbertson et al, 1997), colorectal adenocarcinomas (Ropponen et al, 1999) and in cervical intra-epithelial neoplasms (Hietala et al, 1997). In breast cancer, AP-2α expression was limited to cancer cell nuclei (Turner et al, 1998). Therefore, the pattern of AP-2α expression seems to be tissue type-specific. Other transcription factors such as p53 (Sun et al, 1992, 1995; Bosari et al, 1994; Goldman et al, 1996), BRCA1 (Chen et al, 1995), the C/EBP (CCAAT/enhancer binding protein) (Sundfeldt et al, 1999) and transforming growth factor (TGF)-β inducible early gene (TIEG) (Subramaniam et al, 1998) have also been shown to be expressed in the cancer cell cytoplasm. The precise location of a transcription factor may depend e.g. upon its position in the cell cycle or the phosphorylation status of the factor

**Table 3** Results of Cox's univariate analysis for overall ($n = 290$) survival according to the expression of AP-2α

| Category | Beta (s.e) | Relative risk (95% CI) | *P*-value |
|---|---|---|---|
| Overall survival | | | |
| Nuclear AP-2α expression | | | |
| Negative | * | * | |
| Positive | 0.12 (0.16) | 1.13 (0.83–1.54) | 0.45 |
| Cytoplasmic AP-2α expression | | | |
| Low | 0.45 (0.15) | 1.57 (1.18–2.10) | 0.002 |
| High | * | * | |
| Combined AP-2α expression (nuclear/cytoplasmic) | | | 0.009 |
| –/+ | * | * | |
| +/+ | 0.30 (0.22) | 1.35 (0.88–2.06) | 0.17 |
| +/– | 0.61 (0.28) | 1.85 (1.07–3.20) | 0.03 |
| –/– | 0.57 (0.18) | 1.77 (1.25–2.50) | 0.001 |

*Reference category. CI = confidence interval.

 © 2000 Cancer Research Campaign

**Table 4** Independent prognostic factors in Cox's multivariate analysis for overall (*n* = 261) and recurrence-free (*n* = 148) survival

| Category | Beta (S.E) | Relative risk (95% CI) | *P*-value |
|---|---|---|---|
| Overall survival | | | |
| FIGO stage | | | 0.0009 |
| I | | * | |
| II | 0.53 (0.35) | 1.7 (0.86–3.37) | 0.12 |
| III | 1.03 (0.38) | 2.8 (1.34–5.8) | 0.006 |
| IV | 1.66 (0.43) | 5.3 (2.27–12.3) | 0.0001 |
| Age at diagnosis (years) | | | 0.009 |
| <50 | | * | |
| 50–65 | 0.53 (0.24) | 1.7 (1.05–2.73) | 0.03 |
| >65 | 0.75 (0.25) | 2.13 (1.32–3.44) | 0.002 |
| Primary residual tumour | | | 0.0001 |
| None | | * | |
| ≤2 cm | 1.07 (0.32) | 2.92 (1.56–5.44) | 0.0008 |
| >2 cm | 1.39 (0.32) | 4.03 (2.16–7.54) | <0.00005 |
| Adjuvant chemotherapy | | | 0.007 |
| Platinum-containing | | * | |
| No-platinum containing | 0.40 (0.18) | 1.5 (1.04–2.13) | 0.029 |
| None | 0.91 (0.32) | 2.48 (1.32–4.64) | 0.0046 |
| Cytoplasmic AP-2α expression | | | |
| Low | 0.45 (0.17) | 1.6 (1.13–2.18) | 0.007 |
| High | | * | |
| Recurrence-free survival | | | |
| Primary residual tumour | | | <0.00005 |
| None | | * | |
| ≤2 cm | 1.03 (0.31) | 2.79 (1.52–5.13) | 0.0009 |
| > 2 cm | 1.57 (0.32) | 4.8 (2.56–9.04) | <0.00005 |

*Reference category. CI = confidence interval.

(Karin and Hunter, 1995; Casey, 1997). In addition, cytoplasmic expression may actually represent the transcription factor in a latent form which is ready to be translocated into the nucleus upon activation (Karin and Hunter, 1995). The present study showed the expression of AP-2α to be mostly cytoplasmic but failed to answer why ovarian carcinomas produce this particular transcription factor and what regulates its translocation to the nucleus.

The observed amount of immunohistochemical AP-2α positivity was rather low in the present series. However, even after a very thorough adjustment of the staining conditions inflammatory cells remained positive for AP-2α in tumour sections throughout the staining series verifying the quality of our IHC procedure. We performed mRNA ISH in a subgroup of different tumour types to further elucidate the mechanisms of AP-2α expression. Previously, it has been suggested that the expressional depletion may originate from a chromosomal deletion of AP-2α coding gene (Jean et al, 1998) or post-transcriptional or -translational regulation (Mitchell et al, 1987; Williams et al, 1988). The mRNA for the transactivation domain of AP-2α was actively produced in all cases, yet in 7/19 cases very little protein was detectable by IHC. This result was additionally confirmed by RT-PCR. Changes in cell signalling pathways have been shown to modify protein synthesis (Kleijn et al, 1998), but presently very little is known about the regulation of AP-2α synthesis. However, our results suggest that post-transcriptional regulation may also be one mechanism which controls the expression of AP-2α in ovarian carcinomas.

Based on the previous studies in malignant melanoma (Bar-Eli, 1997; Jean et al, 1998; Karjalainen et al, 1998) and colorectal cancer cell lines (Zeng et al, 1997) AP-2 has been suggested to be a new tumour suppressor gene. The high cytoplasmic AP-2α protein expression without nuclear positivity favoured the survival of epithelial ovarian cancer patients. Similarly, in endometrial cancer, favourable survival associated with cytoplasmic p53 overexpression (Soong et al, 1996). In that and other studies as well, the cytoplasmic expression of p53 has represented mainly functional wild-type protein (Moll et al, 1992; Bosari et al, 1994; Goldman et al, 1996; Soong et al, 1996). Because it is generally accepted that the nuclear location of a transcription factor is essential for its function, the mechanisms by which the cytoplasmic AP-2α expression exerts its prognostic significance in epithelial cancer remain unknown. One possible pathway which may mediate AP-2's growth inhibitory effects is through p21/WAF1 activation documented in human hepatoblastoma and colon adenocarcinoma cells (Zeng et al, 1997). The expressions of nuclear AP-2α and p21/WAF1 did not mutually correlate in the present study group suggesting that p21/WAF1 may not be activated by AP-2α in epithelial ovarian cancer. This is supported by our previous findings which suggested that p21/WAF1 expression is mostly p53-dependent in epithelial ovarian cancer (Anttila et al, 1999).

Interestingly, in survival analyses the combination of nuclear and low cytoplasmic AP-2α expression associated with a significant risk of dying due to ovarian cancer. It may be possible that as the malignant growth gets beyond a certain point or histological type (e.g. serous) there is a shift to a type of expression where AP-2α is needed to activate oncogenes and/or growth factors. Indeed, previous studies have suggested that AP-2 regulates the proto-oncogene c-*erb*B-2 in breast cancer (Bosher et al, 1996) and that it has a role in the function of Ras oncoprotein (Kannan et al, 1994). Similarly, in cervival cancer HPV-16 needs AP-2α to actively produce its own oncogenes E6 and E7 (Bossler AD et al, manuscript

© 2000 Cancer Research Campaign in preparation). Summarizing the available information it is obvious that further studies are needed to extend our knowledge on the regulation of AP-2α expression as well as its function in cancer.

To conclude, AP-2α is expressed in epithelial ovarian carcinomas and its expression is associated with survival of the patients. In particular, the cytoplasmic expression seems to favour a better prognosis. To our surprise, the translocation of AP-2α to its assumed functional place, the nucleus, clearly worsened patients' survival. Whether this is an indication of a cooperation between AP-2α and an oncogene remains to be seen. Finally, our mRNA results suggest that post-transcriptional events may be important in the regulation of AP-2α, but the precise mechanisms mediating AP-2α's effects will still need further exploration in the future.

### ACKNOWLEDGEMENTS

Technical assistance of Ms. Helena Kemiläinen and statistical advice of Ms. Pirjo Halonen is gratefully acknowledged. The financial support of the Special Government Funding of Kuopio University Hospital, Finnish Cancer Foundation, The Finnish Medical Society Duodecim and Paavo Koistinen Foundation is acknowledged.

### REFERENCES

Anttila MA, Kosma VM, Hongxiu J, Puolakka J, Juhola M, Saarikoski S and Syrjanen K (1999) p21/WAF1 expression as related to p53, cell proliferation and prognosis in epithelial ovarian cancer. *Br J Cancer* **79**: 1870–1878

Bar-Eli M (1997) Molecular mechanisms of melanoma metastasis. *J Cell Physiol* **173**: 275–278

Batsche E, Muchardt C, Behrens J, Hurst HC and Cremisi C (1998) RB and c-MYC activate expression of the E-Cadherin gene in epithelial cells through interaction with transcription factor AP-2. *Mol Cell Biol* **18**: 3647–3658

Bosari S, Viale G, Bossi P, Maggioni M, Coggi G, Murray JJ and Lee ACK (1994) Cytoplasmic accumulation of p53 protein: an independent prognostic indicator in colorectal adenocarcinomas. *J Natl Cancer Inst* **86**: 681–687

Bosher JM, Totty NF, Hsuan JJ, Williams T and Hurst HC (1996) A family of AP-2 proteins regulates c-erbB-2 expression in mammary carcinoma. *Oncogene* **13**: 1701–1707

Bosher JM, Williams T and Hurst HC (1995) The developmentally regulated transcription factor AP-2 is involved in c-erbB-2 overexpression in human mammary carcinoma. *Proc Natl Acad Sci USA* **92**: 744–747

Cancer Committee of the International Federation of Gynecology and Obstetrics (1989) Staging Announcement: FIGO Cancer Committee. *Gynecol Oncol* **25**: 383–385

Casey G (1997) The BRCA1 and BRCA2 breast cancer genes. *Curr Opin Oncol* **9**: 88–93

Chen Y, Chen CF, Riley DJ, Allred DC, Chen PL, Von Hoff D, Osborne CK and Lee WH (1995) Aberrant subcellular localization of BRCA1 in breast cancer. *Science* **270**: 789–791

Chiu R, Imagawa M, Imbra RJ, Bockoven JR and Karin M (1987) Multiple cis- and trans-acting elements mediate the transcriptional response to phorbol esters. *Nature* **329**: 648–651

Cox DR (1999) Regression models and life tables with discussion. *J Stat Soc B* **34**: 187–192

Foulkes WD, Ragoussis J, Stamp GWH, Allan GJ and Trowsdale J (1993) Frequent loss of heterozygosity on chromosome 6 in human ovarian carcinoma. *Br J Cancer* **67**: 551–559

Gilbertson RJ, Perry RH, Kelly PJ, Pearson AD and Lunec J (1997) Prognostic significance of HER2 and HER4 coexpression in childhood medulloblastoma. *Cancer Res* **57**: 3272–3280

Goldman SC, Chen CY, Lansing TJ, Gilmer TM and Kastan MB (1996) The p53 signal transduction pathway is intact in human neuroblastoma despite cytoplasmic localization. *Am J Pathol* **148**: 1381–1385

Hennig G, Lowrick O, Birchmeier W and Behrens J (1996) Mechanisms identified in the transcriptional control of epithelial gene expression. *J Biol Chem* **271**: 595–602

Hietala KA, Kosma VM, Syrjanen KJ, Syrjanen SM and Kellokoski JK (1997) Correlation of MIB-1 antigen expression with transcription factors Skn- 1, Oct-1, AP-2, and HPV type in cervical intraepithelial neoplasia. *J Pathol* **183**: 305–310

Huhtala P, Chow LT and Tryggvason K (1990) Structure of the human type IV collagenase gene. *J Biol Chem* **265**: 11077–11082

Hyman SE, Comb M, Pearlberg J and Goodman HM (1989) An AP-2 element acts synenergistically with the cyclic AMP- and phorbol ester-inducible enhancer of the human proenkephalin gene. *Mol Cell Biol* **9**: 321–324

Imagawa M, Chiu R and Karin M (1987) Transcription factor AP-2 mediates induction by two different signal-transduction pathways: protein kinase C and cAMP. *Cell* **51**: 251–260

Ito Y, Kobayashi T, Takeda T, Komoike Y, Wakasugi E, Tamaki Y, Tsujimoto M, Matsuura N and Monden M (1996) Expression of p21 (WAF1/CIP1) protein in clinical thyroid tissues. *Br J Cancer* **74**: 1269–1274

Jean D, Gershenwald JE, Huang S, Luca M, Hudson MJ, Tainsky MA and Bar-Eli M (1998) Loss of AP-2 results in up-regulation of MCAM/MUC18 and an increase in tumor growth and metastasis of human melanoma cells. *J Biol Chem* **273**: 16501–16508

Kannan P, Buettner R, Chiao PJ, Yim SO, Sarkiss M and Tainsky MA (1994) N-ras oncogene causes AP-2 transcriptional self-interference, which leads to transformation. *Genes Dev* **8**: 1258–1269

Kaplan EL and Meier P (1958) Non-parametric estimation from incomplete observation. *J Am Stat Assoc* **53**: 457–481

Karin M and Hunter T (1995) Transcriptional control by protein phosphorylation: signal transmission from the cell surface to the nucleus. *Curr Biol* **7**: 747–757

Karjalainen JM, Kellokoski JK, Eskelinen MJ, Alhava EM and Kosma VM (1998) Downregulation of transcription factor AP-2 predicts poor survival in stage I cutaneous malignant melanoma. *J Clin Oncol* **16**: 3584–3591

Kleijn M, Scheper GC, Voorma HO and Thomas AAM (1998) Regulation of translation initiation factors by signal transduction. *Eur J Biochem* **253**: 531–544

Leask A, Byrne C and Fuchs E (1991) Transcription factor AP-2 and its role in epidermal-spesific gene expression. *Proc Natl Acad Sci USA* **88**: 7948–7952

Lee W, Haslinger A, Karin M and Tjian R (1987) Activation of transciption by two factors that bind promoter and enhancer sequences of the human metallothionein gene and SV40. *Nature* **325**: 368–372

Luscher B, Mitchell PJ, Williams T and Tjian R (1989) Regulation of transcription factor AP-2 by the morphogen retinoic acid and by second messengers. *Genes Dev* **3**: 1507–1517

Mitchell PJ, Wang C and Tjian R (1987) Positive and negative regulation of transcription in vitro: enhancer-binding protein AP-2 is inhibited by SV40 T antigen. *Cell* **50**: 847–861

Mitchell PJ, Timmons PM, Hebert JM, Rigby PW and Tjian R (1991) Transcription factor AP-2 is expressed in neural crest cell lineages during mouse embryogenesis. *Genes Dev* **5**: 105–119

Moll UM, Riou G and Levine AJ (1992) Two distinct mechanisms alter p53 in breast cancer: mutation and nuclear exclusion. *Proc Natl Acad Sci USA* **89**: 7262–7266

Ropponen KM, Kellokoski JK, Lipponen PK, Pietiläinen T, Eskelinen MJ, Alhava EM and Kosma VM (1999) p21/WAF1 expression in human colorectal carcinoma: association with p53, transcription factor AP-2 and prognosis. *Br J Cancer*, **81**: 133–140

Sato M, Saito H, Morita R, Koi S, Lee JH and Nakamura Y (1991) Allelotype of human ovarian cancer. *Cancer Res* **51**: 5118–5122

Serov SF, Scully R and Sobin LH (1973) Histological typing of ovarian tumours. *International Histological Classification of Tumours* no. **9**. Geneva: WHO

Soong R, Knowles S, Williams KE, Hammond IG, Wysocki SJ and Iacopetta BJ (1996) Overexpression of p53 protein is an independent prognostic indicator in human endometrial carcinoma. *Br J Cancer* **74**: 562–567

Sperry A, Jin L and Lloyd RV (1996) Microwave treatment enhances detection of RNA and DNA by in sity hybridization. *Diagn Mol Pathol* **5**: 291–296

Subramaniam M, Hefferan TE, Tau K, Peus D, Pittelkow M, Jalal S, Riggs BL, Roche P and Spelsberg TC (1998) Tissue, cell type, and breast cancer stage-specific expression of a TGF-beta inducible early transcription factor gene. *J Cell Biochem* **68**: 226–236

Sugg SL, Ezzat S, Rosen IB, Freeman JL and Asa SL (1998) Distinct multiple RET/PTC gene rearrangements in multifocal papillary thyroid neoplasia. *J Clin Endocrinol Metabol* **83**: 4116–4122

Sun XF, Carstensen JM, Zhang H, Stal O, Wingren S, Hatschek T and Nordenskjöld B (1992) Prognostic significance of cytoplasmic p53 oncoprotein in colorectal adenocarcinoma. *Lancet* **340**: 1369–1373

 © 2000 Cancer Research Campaign

Sun X, Shimizu H and Yamamoto K (1995) Identification of a novel p53 promoter element involved in genotoxic stress-inducible p53 gene expression. *Mol Cell Biol* **15**: 4489–4496

Sundfeldt K, Ivarsson K, Carlsson M, Enerback S, Janson PO, Brannstrom M and Hedin L (1999) The expression of CCAAT/enhancer binding protein (C/EBP) in the human ovary in vivo: specific increase in C/EBPbeta during epithelial tumour progression. *Br J Cancer* **79**: 1240–1248

Turner BC, Zhang J, Gumbs AA, Maher MG, Kaplan L, Carter D, Glazer PM, Hurst HC, Haffty BG and Williams T (1998) Expression of AP-2 transcription factors in human breast cancer correlates with the regulation of multiple growth factor signalling pathways. *Cancer Res* **58**: 5466–5472

Wakasugi E, Kobayashi T, Tamaki Y, Ito Y, Miyashiro I, Komoike Y, Takeda T, Shin E, Takatsuka Y, Kikkawa N, Monden T and Monden M (1997) p21(Waf1/Cip1) and p53 protein expression in breast cancer. *Am J Clin Pathol* **107**: 684–691

Williams T, Admon A, Luscher B and Tjian R (1988) Cloning and expression of AP-2, a cell-type-speCific transcription factor that activates inducible enhancer elements. *Genes Dev* **2**: 1557–1569

Williamson JA, Bosher JM, Skinner A, Sheer D, Williams T and Hurst HC (1996) Chromosomal mapping of the human and mouse homologues of two new members of the AP-2 family of transcription factors. *Genomics* **35**: 262–264

Winning RS, Shea LJ, Marcus SJ and Sargent TD (1991) Developmental regulation of transcription factor AP-2 during *Xenopus laevis* embryogenesis. *Nucleic Acids Res* **19**: 3709–3714

Zeng YX, Somasundaram K and el-Deiry WS (1997) AP2 inhibits cancer cell growth and activates p21 WAF1/CIP1 expression. *Nat Genet* **15**: 78–82

*© 2000 Cancer Research Campaign*
